# Assessment of Global Health Education: The Role of Multiple-Choice Questions

**DOI:** 10.3389/fpubh.2021.640204

**Published:** 2021-07-22

**Authors:** Nathan T. Douthit, John Norcini, Keren Mazuz, Michael Alkan, Marie-Therese Feuerstein, A. Mark Clarfield, Tzvi Dwolatzky, Evgeny Solomonov, Igor Waksman, Seema Biswas

**Affiliations:** ^1^Department of Geriatrics, Internal Medicine Residency, East Alabama Medical Center, Opelika, AL, United States; ^2^BMJ Case Reports, London, United Kingdom; ^3^FAIMER, Educational Commission for Foreign Medical Graduates, Philadelphia, PA, United States; ^4^Psychiatry Department, Upstate Medical University, Syracuse, NY, United States; ^5^Anthropology, Hadassah Academic College, Jerusalem, Israel; ^6^Faculty for Health Sciences, Ben Gurion University of the Negev, Be'er Sheva, Israel; ^7^Medical School for International Health, BGU Faculty for Health Sciences, Be'er Sheva, Israel; ^8^Open Clinic, Physicians for Human Rights, Tel Aviv, Israel; ^9^Department of Geriatrics and Centre for Global Health, Faculty of Health Sciences, Ben Gurion University of the Negev, Be'er Sheva, Israel; ^10^Department of Geriatrics, McGill University, Montreal, QC, Canada; ^11^Geriatric Unit, Rambam Health Care Campus, Faculty of Medicine, Technion-Israel Institute of Technology, Haifa, Israel; ^12^General and Hepatobiliary Surgery, Ziv Medical Center, Tzfat, Israel; ^13^The Azrieli Faculty of Medicine, Bar Ilan University, Tzfat, Israel; ^14^Department of Surgery, Galilee Medical Center, Nahariya, Israel

**Keywords:** global health, medical education, multiple-choice questions, assessment, single-best option

## Abstract

**Introduction:** The standardization of global health education and assessment remains a significant issue among global health educators. This paper explores the role of multiple choice questions (MCQs) in global health education: whether MCQs are appropriate in written assessment of what may be perceived to be a broad curriculum packed with fewer facts than biomedical science curricula; what form the MCQs might take; what we want to test; how to select the most appropriate question format; the challenge of quality item-writing; and, which aspects of the curriculum MCQs may be used to assess.

**Materials and Methods:** The Medical School for International Health (MSIH) global health curriculum was blue-printed by content experts and course teachers. A 30-question, 1-h examination was produced after exhaustive item writing and revision by teachers of the course. Reliability, difficulty index and discrimination were calculated and examination results were analyzed using SPSS software.

**Results:** Twenty-nine students sat the 1-h examination. All students passed (scores above 67% - in accordance with University criteria). Twenty-three (77%) questions were found to be easy, 4 (14%) of moderate difficulty, and 3 (9%) difficult (using examinations department difficulty index calculations). Eight questions (27%) were considered discriminatory and 20 (67%) were non-discriminatory according to examinations department calculations and criteria. The reliability score was 0.27.

**Discussion:** Our experience shows that there may be a role for single-best-option (SBO) MCQ assessment in global health education. MCQs may be written that cover the majority of the curriculum. Aspects of the curriculum may be better addressed by non-SBO format MCQs. MCQ assessment might usefully complement other forms of assessment that assess skills, attitude and behavior. Preparation of effective MCQs is an exhaustive process, but high quality MCQs in global health may serve as an important driver of learning.

## Introduction

Multiple Choice Questions (MCQs) are the most commonly used tool for assessment in medical education (1, 2). While other tools including short answer questions, long answer questions, oral examinations, and written reports also have an important role in assessment, the vast majority of summative examinations a medical student takes are based on MCQs (1, 3, 4). The popularity of MCQs rests on the ease of testing a breadth of knowledge, standard setting and production of statistical data necessary for quality control of institutional question banks (4–6). Common criticisms of standard MCQs include their failure to engage higher order thinking, test attitude, behaviors or the application of clinical skills, and their failure to take into account gender and cultural biases in question response. Thus, standard MCQs are not generally associated with transformational learning (4, 5, 7). There are, however, a multitude of MCQ styles that may, to varying degrees, test knowledge, skills, attitudes, judgement and even behavior, especially when questions are context-based.

Examples of MCQ style are given in Table 1 below which summarizes the features of each type of question: single best option (SBO); true/false statements; extended matching; situational judgement; and, script concordance questions (8–11). For the most part, SBO style MCQs are used to assess the biomedical curriculum. This is the case in national licensing exams such as the United States Medical Licensing Examination (12).

**Table 1:** MCQ question types and descriptions.

**Question type**	**Components**	**Adjustments**	**Instructions**
Single best option	A question stem with multiple distractors	Although distractors may be plausible, there is a single best answer to the stem	Choose the single best answer
True or false	A statement	Each statement is either true or false	Mark whether each statement is true or false
Extended matching	Stem, lead in question, options, multiple distractors	The same answer may apply to multiple questions	Select one answer to each question
Situational judgement	Scenario, variety of responses	Each response is rated by the student	Rate the appropriateness of each response
Script concordance	Case, relevant diagnostic/management options and findings, options	The options assess the direction and intensity of new findings on the student's reasoning	Indicate whether the new finding has a positive or negative effect on the hypothesis

In comparison to the techniques used for the assessment of the basic sciences, assessment of global health learning is more problematic. While lectures and written learning resources may be rich in factual content, there is a perception among students, and perhaps even faculty, that these facts are not immediately relevant to a scientific medical curriculum, and that a grasp of global health concepts does not necessarily require a recall of facts (13, 14). Thus, many global health programs rely exclusively on reflective essays as assessment tools—focusing on cultural and anthropological exploration, and ethics and overseas medical experience, or on a project thesis that seeks to address a distinct research question in line with Master of Public Health programs (15–17). This is at odds with standard assessment of the biomedical curriculum which substantially requires the demonstration of recall and understanding of specific facts in order to demonstrate competence to practice.

While many medical schools describe their global health learning programs in detail, there is a paucity of research into what students actually learn on these courses. There is evidence that students know far less than they think they do (13, 14, 18, 19). Eichbaum (20) writes of a frenzied growth in global health education programs with poorly defined goals and objectives, describing the need for competency-based programs. Over the last 10 years there have been calls for an agreement on undergraduate global health education frameworks and competencies (21–28). These competencies are similar to those developed by the Joint US/Canadian Committee on Global Health Core Competencies and those discussed in the 2008 Bellagio conference on global health education and include learning about: the global burden of disease; health implications of travel, migration and displacement; social and economic determinants of health; population resources and environment; globalization of health and health care; health care in low-resource settings; and, human rights in global health. The global health competencies of the Medical School for International Health (MSIH), Ben-Gurion University in Be'er Sheva, Israel, mirror these international standards.

Reflective essays alone cannot test the breadth of such curricula and do not reflect the broad range of global health competencies. Moreover, this style of assessment encourages students to view global health as a “soft” science, less of a priority in learning than the traditional disciplines usually covered in the biomedical curriculum, and to overestimate how much they actually know about global health (13, 14, 29).

Table 2 gives examples of factual learning outcomes in the global health curriculum. Just as the breadth of the biomedical curriculum may be assessed through a SBO MCQ, we propose that for substantial areas of the global health curriculum SBO MCQs may be a useful assessment tool. Table 3 gives examples of how these learning outcomes might be assessed using different MCQ formats.

**Table 2:** Topics routinely tested by MCQ assessment and their relevance in global health.

Topic	Examples of application to Global Health
Definitions	Difference between people trafficking and people smuggling Difference between asylum seeker and refugee
International organizations	The world bank, IMF and international aid programs Millennium and sustainable development goals
SPHERE standards	Testing potable water Standards in hygiene and sanitation
Biostatistics	Diseases contributing to mortality in children under the age of 5 years Trauma and burns in school children
Infectious Disease	Parasite infestation and steps in eradication AIDS pandemic
Health policy	Universal Health Coverage Age-friendly cities


**Table 3:** Examples of global health MCQ assessment questions.

**Example**	**Question**	**Answers**	**Analysis**
1. Local context-linked: public health program in Israel	You have been working with a team of Family Medicine practitioners on a program to reduce the risk of diabetes in a Bedouin community in the Negev. The program aims to educate families in a healthy diet and to measure blood sugar and weight and encourage physical exercise. After 6 months it becomes obvious that the program is failing and that there is very little interest from the community in engaging with the program. Which ONE of the following factors is most likely to be the reason for failure of the program?	A. Advising all women to cook bread with brown whole meal flour B. Dialogue only with Bedouin women C. Failure of your team to perform blood sugar and weight checks more than once in 6 months D. Insufficient and non-transparent funding E. Too many aims to achieve in the program	Analysis of examination results shows that 59% selected the correct answer, 31% option B, 7% option A and 1% option E (discrimination 0.83), none of the MSIH control group selected the correct answer, 40% selected option B, 40% option E, 12% option D and 2 option A (discrimination 0).
2. Global Context linked-disaster response abroad	In the aftermath of a disaster in an agricultural village, SPHERE standards are to supply a minimum of 15 liters of water per person per day as part of the disaster response. According to SPHERE standards which ONE of the following is the most urgent initial priority in water supply?	A. Ensuring that drinking water is free of coliforms B. Producing sufficient water sources in order to avoid queuing for longer than 1 h C. Providing sufficient water for essential livestock D. Reducing the environmental impact of the water sources identified and used E. Reducing the distance of all households to the nearest water point to 5 m	Analysis of examination results shows that all of the study group answered this question correctly (discrimination 0), 64% of the MSIH control group answered correctly, 16% selected option C, 12% option B, 4% option D and 4% option D (discrimination−0.12)

In this research we describe the challenges and limitations of devising MCQs for the assessment of knowledge across the global health curriculum and report our experience of SBOs.

## Materials and Methods

### Context

The Medical School for International Health (MSIH) was founded in 1996 as a collaborative effort between the Ben-Gurion University of the Negev in Be'er Sheva, Israel, and the Columbia University Medical Center in New York. The goal of the medical school is to produce physicians who are competent international health practitioners (30, 31). Students are mainly from the USA, some are from Canada and a few from outside North America. Teaching and assessment are conducted in English.

As part of a first year global health teaching program review at MSIH we sought to assess global health learning: looking specifically to see how many of the curricular learning outcomes may be taught and learned over a two-semester global health course; and how to assess what has been learned. Changes to the course and assessment were gradual—over, at least, 14 months (spanning two taught courses). The number of guest lectures was reduced from previous iterations of the course and student involvement in local community programs and patient interaction increased on practical placements. Material from practical placements was incorporated into lectures so that, in principle, all students had exposure to the same learning objectives within the curriculum. Learning objectives from published global health competencies were mapped to the teaching curriculum. MCQs were chosen as they were already in widespread use across the biomedical curriculum. A 30-question SBO MCQ examination was designed and administered to students at the end of the course.

### Blue Printing the Curriculum

Learning objectives within each section of the global health curriculum were defined and teaching faculty agreed on the weighting of topics within the curriculum in terms of MCQ assessment (Table 4). Teachers prepared learning materials (lectures and discussion topics) with these learning objectives in mind and prepared MCQs based on these objectives.

**Table 4:** MCQ Blueprint for global health learning objectives.

**GH Organizations (5%)**	**Number of Questions**
History	0–1
Current role	1–2
**Health Systems 5%**	
Structures of healthcare systems	1–3
Application of health services management to lower and middle income countries	1–2
Concept and dimensions of health system performance	1–2
National, inter-organizational, community and patient level interventions to improve health systems	1–4
**Health Economics 4%**	
Major financing methods for health care and global health efforts	1–2
Key factors in choosing the type of health care financing system	1–2
Major sources of funding in global health and how resources are allotted	1–2
**Health Policy 5%**	
How health policies are made and implemented	1–2
How data on global health measures affect policy change and development	1–3
**Politics 1%**	
Importance of local and international politics in the delivery and efficacy of global health and medicine	1–2
Different methods and tools that healthcare providers can utilize for political advocacy	1–2
**Global Burden of Disease**	**Number of Questions**
**Determinants of Health 10%**	
Why it is important to measure health and disease	1–2
Composite measures of burden of disease, their relative strengths and weaknesses, and how they are used in public health literature, World Health Organization reports	3–5
Understanding of poverty and global health inequalities	2–4
**Global Patterns of Morbidity and Mortality 3%**	
Measures of morbidity and mortality used globally	0–1
Interpretation of tests and how they apply to global health	0–1
**Epidemiology, Biostatistics and Surveillance 2%**	
Measures of morbidity and mortality used globally	0–1
Interpretation of tests and how they apply to global health	0–1
**Infectious and Chronic Disease 5%**	
Epidemiology of various diseases and the threat they pose to health around the world	0–1
Leading causes of morbidity and mortality around the world in low, middle and high income countries	1–2
Describe nutrition problems around the world	0–1
Describe key interventions for malnutrition settings	1–2
Understand the global impact of injuries	0–1
Understand trends and changes in chronic disease incidence	0–1
Identify reasons for changes in chronic disease incidence and prevalence	0–1
Know the range of prevention and treatment strategies for chronic diseases in a range of international settings	0–1
**Environmental Health 5%**	
Understand how geography and climate of a region can impact human health	1–2
Understand the importance of environmental issues such as pollution, natural disasters and climate change and the impact they have on health	1–2
**Cross Cultural Medicine**	**Number of Questions**
**Cultural Sensitivity 1%**	
Role that culture plays in the practice of medicine and global health	0–1
Culturally distinct beliefs, attitudes and practices relating to health and medicine	0–1
**Medical Anthropology 4%**	
Practice and theory of medical anthropology and its role in global health and medicine	2–3
**Vulnerable Populations**	**Number of Questions**
**Maternal and Child Health 4%**	
Social and economic context of maternal and child health	1–2
Basic terms and definitions of indicators specific to these populations	1–2
Main causes of morbidity and mortality for mothers, neonates, infants, and children	1–2
Maternal health issues and interventions as distinct from other women's health issues	1–2
Low-cost, effective, community-based approaches to intervention	1–2
**Disasters, Displaced Persons, Refugees 4%**	
Principles and laws governing international humanitarian assistance	1–2
Basic needs for human survival including water, food, sanitation and safety	1–2
Most common causes of morbidity and mortality in populations affected by conflict, disaster	1–2
Key assessment strategies and public health interventions in disaster management	1–2
Human rights legislation and enforcement	1–3
**Aging Populations 4%**	
Demography of global aging and its relationship to non-communicable diseases	1–2
How the expanding elderly population will influence global health in the future	1–2
**Mental Health 4%**	
Epidemiology and impact of mental health issues on populations	1–2
Economic and social costs of mental illness on populations	1–2
Relationship between mental health and chronic illness	1–2
Barriers to effective treatment of mental illness	1–2
**Poverty 4%**	
How poverty can affect health and how health problems can result in poverty	1–2
How both absolute and relative poverty act as key determinants of health	1–2
**Primary Care Medicine**	**Number of Questions**
**Primary care in global health 4%**	
Define primary care and understand the way it is defined and practiced in different cultures and health systems	1–2
Understand the history of primary care and its global importance today	1–2
Recognize how countries with inadequate primary healthcare are adversely affected and how stressors are manifest on primary healthcare providers	1–2
Identify the various ways in which access to primary care can be blocked or facilitated	1–2
**Preventative Medicine 4%**	
Identify strategies and goals of health systems to prevent illness, including education, screening, vaccination and prophylaxis	1–2
Be familiar with current trends in national and international prevention programs for infectious and chronic illness.	1–2
**Global Pediatrics 4%**	
Understand the unique health needs of infants, children and adolescents	1–2
Be familiar with organizations and programs geared toward child health	1–2
Be familiar with global pediatric vaccination recommendations	1–2
**Sexual and Reproductive Health 4%**	
Understand the unique health needs of infants, children and adolescents	1–2
Be familiar with organizations and programs geared toward child health	1–2
Be familiar with global pediatric vaccination recommendations	1–2
**Access to Essential Medicines 4%**	
Understand the current definition and suggested list of essential medications as defined by the WHO and Doctors Without Borders	1–2
Be familiar with current efforts and limitations to increase global access to essential medications	1–2
**Global Health Ethics**	**Number of Questions**
**Global Health Equity 2%**	
Describe the meaning of the right to health and understand the concept of social justice	0–1
Describe the difference between equity and equality	0–1
Describe the major factors in global health disparities	0–1
**Equity in knowledge sharing 2%**	
Understand the ethical reasoning for open source resources	0–1
Understand the concept and rationale for open-source journals	0–1
Understand the technical aspects of open-access technology including mobile data collection and medical records	0–1

### Item Writing and Testing

MCQs were written by all (four) teachers of the course who taught distinct parts of the curriculum. The SBO format was chosen over the other MCQ styles as there was only one correct answer per question, there was broader agreement on a single correct answer, and questions were deemed less susceptible to guessing. Each question focused on a single learning objective. The final 30 questions chosen out of 100 authored by all teachers of the course were agreed to represent a broad representation of the course material.

The 30 questions were chosen after exhaustive item review. Items were discussed and tested with the faculty (seven teachers from MSIH and four teachers from other faculties), independently (forty medical students in the United Kingdom and seven students in Israel at a different medical school). Criteria for agreement on the final 30 questions comprising the examination were that each question had a meaningful stem free of irrelevant detail, the stem ended with a question, negative phrasing was not used, and that distractors were clear, concise, roughly the same number of words, mutually exclusive and independent of each other. Distractors included common misconceptions discussed in class and were plausible alternatives unless students' precise knowledge of the topics was tested. Any questions with “all/none of the above” or non-heterogeneous distractors were omitted or rewritten. Distractors were listed in alphabetical order.

### Data Analysis

Item analysis and exam statistics provided information about the quality of MCQs and difficulty and discrimination index. The aim was to develop questions that would principally test the recall of facts (in particular definitions, criteria and structural frameworks used in global health as in Table 2).

Reliability was based on Kuder-Richardson 21 (testing reliability of binary variables—where an answer is right or wrong when questions do not vary widely in their level of difficulty). We assumed all questions were equal in difficulty and the binary variable was a correct or incorrect answer. Difficulty-index was calculated as follows:

$$Difficulty\;Index=\frac{Number\;of\;students\;who\;answered\;correctly}{Number\;of\;students\;who\;answered}\times 100$$

Discrimination was computed by comparing students with the highest score to students with the lowest score.

$$Discrimination=2\times (\frac{Number\;of\;students\;in\;highest\;group\;with\;correct\;answer-number\;of\;students\;in\;lowestgroup\;with\;correct\;answer}{Total\;number\;of\;students\;in\;both\;groups})$$

## Results

Examples of multiple-choice questions used in the 30-question examination are seen in Table 3. The MSIH Examinations Department administered and analyzed student performance in the examination using their standard statistical tools and WHO guidelines. One-way ANOVA was applied to detect differences between the student scores using Statistical Product and Service Solutions (SPSS) software. This software was also used to determine discrimination, difficulty and reliability.

Table 5 shows the examination statistics for the 30-question examination. Twenty-nine students sat the 1-h examination. All students passed (scores above 67% as determined by MSIH examinations criteria). Twenty-three (77%) questions were found to be easy, 4 (14%) of moderate difficulty, and 3 (9%) difficult according to the examinations office statistical criteria. Eight questions (27%) were considered discriminatory and 20 (67%) were non-discriminatory. The reliability score was 0.27.

**Table 5:** Statistics of administered global health examinations.

	**Examination statistics from 30-question MCQ paper**
Average	84
Median	85
Standard Deviation	6.3
Reliability	0.27
Number of questions	30
Number of students	29
Students passing (>65)	29
Excellent scores (>90)	0
Failed (<65)	0
Minimum–maximum score	70–97
**Difficulty of Questions**	
Easy (90–100)	20 (67%)
Easy (80–89)	2 (7%)
Easy (70–79)	1 (3%)
Moderate (60–69)	3 (10%)
Moderate (50–59)	1 (3%)
Difficult (40–49)	1 (3%)
Difficult (30–39)	1 (3%)
Difficult (<29)	1 (3%)
**Distribution of Discrimination**	
High (>0.35)	3 (10%)
Medium (0.25–0.34)	2 (7%)
Low (0.15–0.24)	3 (10%)
Non-discriminating (<0.14)	20 (67%)
Negative (<0)	2 (7%)

## Discussion

### The Role of MCQ in Global Health Assessment

The function of assessment has been described as maximizing student competence while guiding subsequent learning. Multiple assessment methods are needed to test all aspects of competence (32). Assessment (or practice for assessment) drives learning. Our experience indicates that identifying ‘factual' aspects of the global health curriculum is possible and that SBO MCQs may be tailored to assess recall and application of these facts. As factual aspects are spread across the curriculum (Table 4), global health MCQs may be employed to assess learning of the breadth of the curriculum just as in the basic sciences. Particular “facts” include definitions, roles of organizations, epidemiological trends in disease prevalence and agreed global standards or health-related goals (Table 2).

We believe that introducing MCQ assessment into a global health curriculum may introduce students to the perception of global health learning as a “hard” science with knowledge and skill competencies in common with the rest of the standard biomedical curriculum. Further, we believe that self-assessment using MCQs may assist students in defining their own learning needs and identify deficiencies in knowledge and competency for practice.

**Figure 1:**
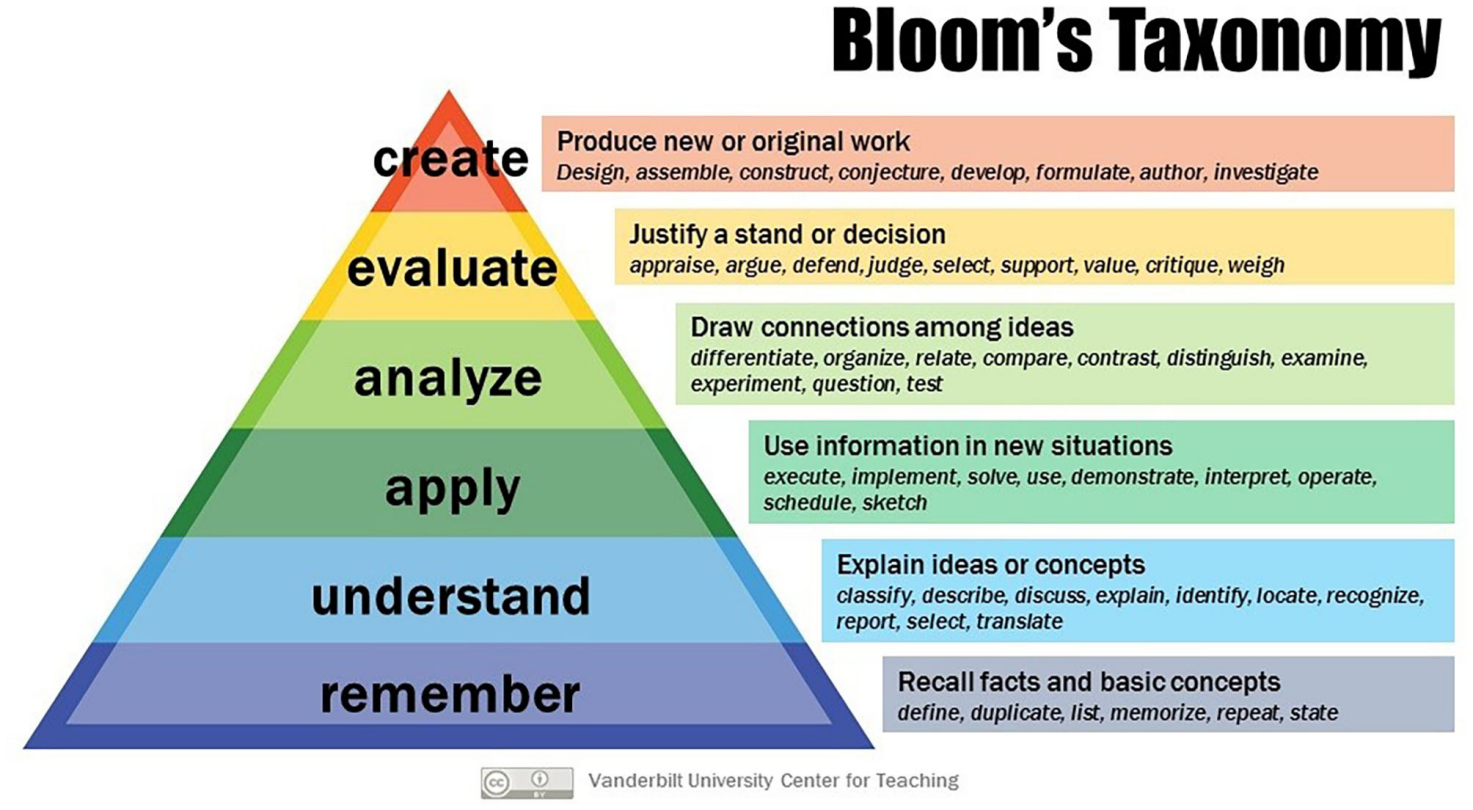
Bloom's taxonomy [Creative Commons, Vanderbilt University. Bloom's Taxonomy. (internet) Vanderbilt University (36). Obtained from https://cft.vanderbilt.edu/guides-sub-pages/blooms-taxonomy/ on 1/3/2020].

In combination with other assessment modalities based on patient-focused assessment tools such as the global health case report, the MCQ may have a role to play in global health education (33–35). Indeed, MCQs may focus learning in what some students may perceive a rather nebulous and unfocused subject. According to Bloom's taxonomy (Figure 1) MCQ-based assessment may overly emphasize the bottom two phases, “remember,” and “understand,” while neglecting higher orders of learning. Topic selection and item writing, therefore, require careful thought that encourages critical (and reflective) thinking, where possible—using diverse MCQ formats (Table 3) (4, 37–39).

### Topics Suitable for MCQ

The suitability of SBO MCQs in assessing breadth of knowledge is relevant in global health assessment (4, 40). Few medical fields require as broad a knowledge base as global health. Topics suitable for MCQ assessment must be identified with clear learning and assessment objectives in mind. The process of defining learning objectives, blueprinting, developing the practical course and putting together a final examination to review took 14 months. We were at pains to ensure that we had taught what we planned to assess and assessed what we knew we had taught. Particular emphasis was placed on understanding why distinct definitions exist to describe vulnerable populations and the rights and entitlements these entail, for example (Table 2). While students may feel that they have a grasp of these topics, choosing only one option from a list of distractors forces the student to accept that precise knowledge and understanding of the topics is required to practice safely and access the care their patients require. Table 6 offers an example of a question that we believe tests the application of knowledge and understanding of the social determinants of health.

**Table 6:** MCQ example testing understanding and application.

Israel' National Health Insurance Law came into effect in 1995. This entitles refugees, migrants and asylum seekers to emergency health care. The influence of this legislation on public health is an example of a/an ____________ impacting a _________________ of health inequality.	
A.	Downstream social determinant of health; structural determinant
B.	Downstream social determinant of health; intermediary determinant
C.	Upstream social determinant of health; structural determinant
D.	Upstream social determinant of health; intermediary determinant
E.	Upstream social determinant of health; exceptional determinant

### Item Writing and Quality Control

Writing “good” global health questions is a challenge that requires multiple contributors of questions, exhaustive criticism and review. MCQs should be based on a blueprint of the curriculum and test topics most suited to MCQ-style assessment. We emphasized context as students were encouraged to learn global health on practical placements as well as lectures (41). Our aim is to better engage students (providing real-life experience of global health problems patients experience) and make assessment relevant to future clinical practice. Epstein (6) explains that “questions with rich descriptions of the clinical context invite the more complex cognitive processes that are characteristic of clinical practice. Conversely, context-poor questions can test basic factual knowledge but not its transferability to real clinical problems.” Thus, we believe that context-based questions may place potentially abstract global health concepts in practical, realistic settings relevant to students, easier to identify, recall, and apply knowledge.

Although reliability may have been improved by increasing the number of test items in the exam, we opted for a 30-question examination so that the assessment would be completed within 1 h. We selected 30 questions that represented the breadth of the curriculum and our blueprint. A greater spread of scores with more moderately difficult questions (Difficulty Index 0.4–0.8) and discriminatory questions, would also have been preferable.

### Limitations

The most obvious deficiency in this research is that the examination was run only once, and exam questions subjected to statistical analysis only once. This substantially limits any conclusion regarding reliability and validity. Clearly testing needs to be repeated. The research period (a 3-year PhD program that included the running of the global health course) precluded repeated testing. Also important is the development of other styles of MCQs to evaluate their role in the assessment of attitudes and behaviors expected of global health practitioners. We have since increased our bank of questions and written MCQs in other formats better suited to testing judgement, decision making and situational awareness. Examples are given in Table 7. Further testing and analysis of all formats of MCQs for global health teaching and learning is planned.

**Table 7:** Global health examination questions in distinct MCQ formats.

**Single Best Option**	Implementing your programme of multi-drug resistant tuberculosis treatment in the city, an independent public health team finds that after 2 years of your programme there is no obvious change in prevalence of the disease. This is difficult to understand as the programme provides medication for free and health-workers reach out to the population via house calls and visits to places of work and schools in a well-accepted programme. Which ONE of the following is the most likely explanation for a lack of impact of the programme? A. Employers are unwilling to participate in programmes where staff may need to be isolated B.Schools are unwilling to participate in programmes where children may require medication C.Stigma prevents patients in need of treatment coming forward D.There is no outreach programme for the homeless E.The number of households in the city has doubled in the last year							
True or False	Which of the following statements is true of the fundamental aims of Universal Health Coverage? A.Accountability in health care spending B.Commitment to a national health budget in line with local health costs C.Free healthcare for the most vulnerable D.Health care delivery prioritized according to population need E.Setting minimum standards in health care for all							
Extended Matching	Theme: A. Social accountability B.Social capital C.Social inclusion D.Social justice E.Social welfare For each situation below select the most appropriate social construct Question 1 Small holding farmers in 5 neighboring villages form a co-operative to set pricing on sales to local markets B Question 2 Medical schools revise their curricula to include their commitment to local community needs A							
Situational Judgement	You accompany the parents to an outpatient appointment for their 7-year-old child with cystic fibrosis. You know the family well and have been following up the child in the community with home visits as part of your global health course. You get along well with the child and his parents. The doctor asks whether the child has been eating in accordance with the dietary advice given to the parents several months ago. You know that mealtimes are a challenge and most of the time, the only food the child will eat is a small piece of pizza. The parents nod strongly in agreement that they have been sticking to the diet and the doctor adds that she can see that the child looks well although he is below the 75th percentile in weight. Please rank your potential actions in order or appropriateness with 1 being the most appropriate and 4 being the least appropriate. A.You keep quiet during the consultation as the child seems to be well regardless of any particular diet B.You keep quiet but raise the issue politely with the family once you have left the consultation room C.You politely joke during the consultation that mealtimes are such a challenge, most of the time the pizza is the only food the child will eat D.You arrange to meet the doctor later to express your concerns about the child's diet							
Script Concordance	You have been working with people living in a village near a river who have reported becoming ill after drinking water believed to be contaminated by a leak of detergent reported by a nearby detergent factory 4 weeks earlier. The leak was repaired within 24 h of discovery by the factory owners.							
		**If you were thinking that**	**And you find that**	**This Hypothesis becomes**				
	A.	Detergent leaked into the river from the factory	The factory is downstream of the village	−2	−1	0	+1	+2
	B.	Drinking water in the village should be filtered	There are no microorganisms in the community water tank of the village	−2	−1	0	+1	+2
	C.	The leak has caused detergent to contaminate the river	Fish in the river near the factory were found dead 4 weeks ago	−2	−1	0	+1	+2
	D.	River water is contaminated by dead fish	There are no reports of illness in villages upstream of the river in the last 4 weeks	−2	−1	0	+1	+2
	E.	Detergent is present in harmful concentrations in the river 4 weeks after the leak was repaired	Samples of river water tested 3 weeks ago after the leak was repaired were taken from shallow water near the river bank	−2	−1	0	+1	+2

### Conclusion

Global health curricula now have internationally agreed defined competencies and learning objectives (42). As medical teaching worldwide becomes increasingly standardized, there is a need to define precise measures of assessment of student learning that may be used in combination with existing assessment tools such as reflective essays or case reports. We propose further development of MCQs in their diverse formats and testing so that we may determine, not simply their utility in testing what has been learned, but how this knowledge may be applied in the practice of doctors who study global health.

## Data Availability Statement

The raw data supporting the conclusions of this article will be made available by the authors without undue reservation.

## Ethics Statement

The studies involving human participants were reviewed and approved by Ben Gurion University IRB. The patients/participants provided their written informed consent to participate in this study.

## Author Contributions

SB designed and researched the material. SB and ND wrote the article. SB, KM, MA, and M-TF wrote the questions. JN, AC, TD, ES, and IW evaluated the questions. All co-authors were involved in revising the article for important intellectual content and gave final approval of the version to be published.

## Conflict of Interest

SB is editor-in-chief of BMJ Case Reports which publishes Global Health case reports. ND is an associate editor of BMJ Case Reports. The remaining authors declare that the research was conducted in the absence of any commercial or financial relationships that could be construed as a potential conflict of interest.

## Publisher's Note

All claims expressed in this article are solely those of the authors and do not necessarily represent those of their affiliated organizations, or those of the publisher, the editors and the reviewers. Any product that may be evaluated in this article, or claim that may be made by its manufacturer, is not guaranteed or endorsed by the publisher.
